# Adherence to hunger training using blood glucose monitoring: a feasibility study

**DOI:** 10.1186/s12986-015-0017-2

**Published:** 2015-06-09

**Authors:** M. R. Jospe, R. C. Brown, M. Roy, R. W. Taylor

**Affiliations:** Department of Nutrition, University of Otago, Dunedin, New Zealand; Department of Medicine, University of Otago, PO Box 56, Dunedin, 9054 New Zealand

**Keywords:** Food intake regulation, Hunger, Obesity, Blood glucose self-monitoring, Feasibility study, Adherence

## Abstract

**Background:**

“Hunger training”, which aims to teach people to eat only when blood glucose is below a set target, appears promising as a weight loss strategy. As the ability of participants to adhere to the rigorous protocol has been insufficiently described, we sought to determine the feasibility of hunger training, in terms of retention in the study, adherence to measuring blood glucose, and eating only when blood glucose concentrations are below a set level of 4.7 mmol/L.

**Method:**

We undertook a two-week feasibility study, utilising an adaptive design approach where the specific blood glucose cut-off was the adaptive feature. A blood glucose cut-off of 4.7 mmol/L (protocol A) was used for the first 20 participants. *A priori* we decided that if interim analysis revealed that this cut-off did not meet our feasibility criteria, the remaining ten participants would use an individualised cut-off based on their fasting glucose concentrations (protocol B).

**Results:**

Retention of the participants in the study was 97 % (28/29 participants), achieving our criterion of 85 %. Participants measured their blood glucose before 94 % (95 % CI 91, 98) of eating occasions (criterion 80 %). However, participants following protocol A, which used a standard blood glucose cut-off of 4.7 mmol/L, were only able to adhere to eating when blood glucose was below the prescribed level 66 % of the time, below our within-person criterion of 75 %. By contrast, those participants following protocol B (individualised cut-off) adhered to the eating protocol 84 % of the time, a significant (*p* = 0.010) improvement over protocol A.

**Conclusion:**

Hunger training appears to be a feasible method, at least in the short-term, when an individualised fasting blood glucose is used to indicate that a meal can begin.

## Background

The persistent obesity epidemic has generated a plethora of weight loss studies that investigate the effectiveness of diets varying in macronutrient recommendations. However, it appears that modifying the composition of diets has a minor impact on weight loss, especially over six months or longer [1–5]. Instead, a far more relevant factor appears to be the degree of *adherence* to the prescribed diet [1, 6, 7], which may be encouraged by behavioural strategies [8].

One effective strategy for weight loss may be learning to eat only when hungry, as eating in response to environmental, social, or emotional cues rather than physical hunger has been consistently associated with a higher body mass index (BMI) and energy intake [9–11]. Ciampolini et al. [12] have developed an intriguing protocol that shows promise in training people to eat according to their hunger. Participants are trained to connect their physical symptoms of hunger with their blood glucose, and to eat only when their blood glucose is below a set target of 4.7 mmol/L. When tested in a group of 74 overweight participants, this method of “hunger training” (or “hunger recognition”, as coined by Ciampolini et al. [12]) produced significantly greater weight loss over 5 months (3.5 kg), compared with that observed following the conventional approach of increasing vegetable intake and physical activity [13].

While these initial results appear promising, replication by another group would provide further support for the use of this weight management strategy. The upcoming SWIFT trial will test the effectiveness of four behavioural strategies, including hunger training, on adherence to diets and the resulting weight loss in overweight adults during a two-year randomised controlled trial (RCT) [14]. However, we believed there were several important considerations regarding the feasibility of the hunger training protocol that required addressing before we could incorporate it into our larger trial. Firstly, adherence to the protocol does not appear to have been reported, but is critical given that even those who need to self-monitor their blood glucose for diabetes management struggle to do so even once per day [15]. Secondly, some concern was raised regarding the suitability of 4.7 mmol/L as a standard cut-off for all participants given that this is below the fasting glucose level for the majority of non-diabetic adults [16, 17]. Lastly, there was some doubt over whether blood glucose is an appropriate measure of perceived hunger, given that different studies have indicated that blood glucose and hunger are not correlated [18], whereas others [19] have reported significant correlations as high as r = 0.55-0.63.

Given our concerns, we decided to undertake a feasibility study [20] before including hunger training as a support strategy in the larger SWIFT trial. We sought to answer questions about adherence, the use of 4.7 mmol/L as a cut-off, and the appropriateness of using blood glucose to indicate hunger. We used an adaptive design method [21], with the blood glucose cut-off as the adaptive feature [22] to increase our chance of a finding a viable method for hunger training. Adaptive design trials are formulated to allow modification to the study while it is underway, in order to efficiently learn from the data being gathered. *A priori* rules guide the modification of the adaptive feature (the characteristic of the protocol and study which may require modification), and decisions are made based on cumulative data from interim analyses [21, 22]. For this study, we decided *a priori* to analyse the adherence data to the original protocol (protocol A) after 20 participants, before deciding on modifications to the blood glucose cut-off based on predefined feasibility criteria.

The aim of our single-centre, single-arm study was to determine the feasibility of the hunger training protocol [12]–with particular attention to study retention, adherence of measuring blood glucose, and adherence to eating below the blood glucose cut-off-to inform the design and use of hunger training within the large randomised controlled SWIFT trial [14].

## Methods

### Participants and setting

Participants were recruited by advertisement and word of mouth in Dunedin, New Zealand. Eligible participants of both sexes had to be at least 18 years of age. We recruited both normal weight and overweight participants, aiming for a final ratio of 2:1 (overweight: normal weight). While only overweight participants will be included in the main SWIFT trial, normal weight participants were included in this feasibility study primarily to determine whether the results vary with BMI and whether hunger training might be suitable for weight maintenance in normal weight participants, as was suggested in the original protocol [13]. Exclusion criteria included having type 1 or type 2 diabetes or heart disease. Participants had to be willing to measure their blood glucose via finger prick test three to eight times per day, and to wear a continuous glucose monitoring system if required. Participants were informed about the experimental feasibility design before written informed consent was obtained. The study was approved by the University of Otago Human Ethics Committee (H14/076).

### Intervention

**Fig. 1:**
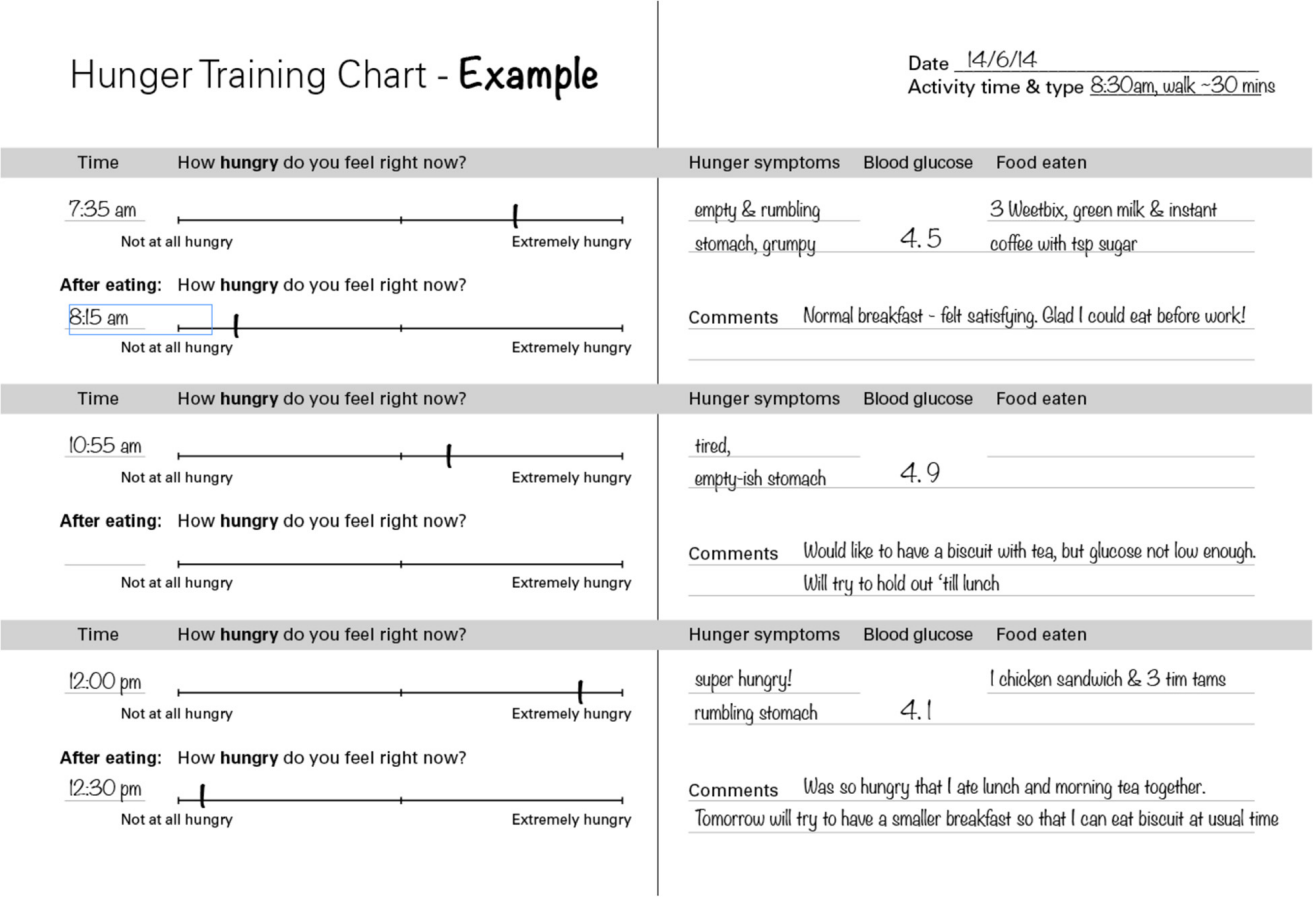
The example page in the hunger training booklet

**Fig. 2:**
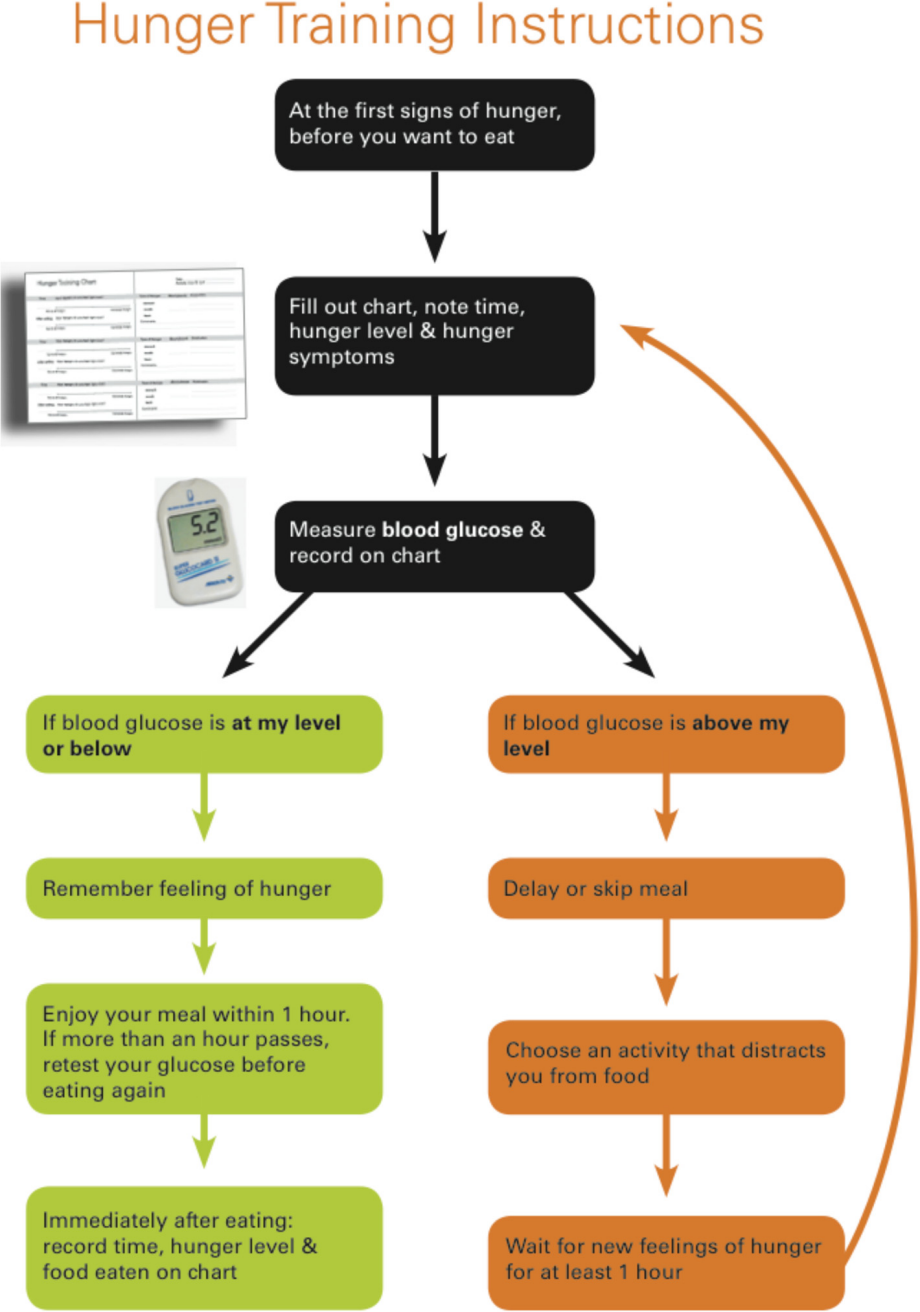
Hunger training instructions in the hunger training booklet

Participants followed the hunger training procedure for two weeks, which was based on Ciampolini et al. [13]. Participants were provided with a booklet in which to record hunger levels, blood glucose values, and food consumed (Fig. 1). Every time a participant wanted to eat, they were instructed to assess their hunger level using a visual analogue scale ranging from 0 mm (not at all hungry) to 100 mm (extremely hungry) [23, 24]. Once that was completed, participants were instructed to measure capillary blood glucose from a finger prick sample by portable glucometer (Abbott Freestyle Optium Glucose Meter, Australia). Participants were only permitted to eat if their blood glucose was under their specified cut-off. If their blood glucose was above their cut-off, they were instructed to choose an activity that distracted them from food, and to wait for new feelings of hunger for at least an hour before testing their blood glucose again (Fig. 2). Participants were permitted to consume hypocaloric drinks at any time. Participants were also specifically instructed that they could only drink alcohol if their blood glucose was below their cut-off.

We suggested that participants eat primarily fruit and vegetables for the first few days of hunger training as per Ciampolini et al. [13] in order to experience hunger more frequently and therefore become more familiar with the symptoms of hunger. As the training progressed, we encouraged participants to gradually reintroduce their typical foods and pay attention to the effect on hunger and blood glucose.

#### Blood glucose Cut-off

To test the appropriateness and feasibility of the blood glucose cut-off, we used an adaptive design approach [21], with blood glucose cut-off as the adaptive feature [22]. *A priori* we decided to initially use 4.7 mmol/L as the cut-off (protocol A) as per the original protocol [12] and to undertake an interim analysis after the first 20 participants had completed the training. If this analysis indicated that the average within-person proportion of eating occasions where blood glucose was below the cut-off was more than 75 %, the cut-off would be individualised following protocol B.

### Protocol A

All participants in cohort A could only eat if their blood glucose was 4.7 mmol/L or less.

### Protocol B

Each participant in cohort B had an individual blood glucose cut-off, which was calculated as the average of the fasting glucose of the first two days of hunger training. If a participant’s individualised cut-off was less than 4.7 mmol/L, they were given a cut-off of 4.7 mmol/L. During the first two days of hunger training, participants used their fasting glucose of that morning for their cut-off for the day.

### Procedure

Participants met with the researcher on three occasions over two weeks (baseline, day 7 and day 14). At the first visit, height was measured with a fixed stadiometer (Heightronic, QuickMedical, WA, USA) and weight by electronic scales (Tanita BC-418). Participants completed a brief questionnaire that included demographic information [25], the self-administered short form of the International Physical Activity Questionnaire (IPAQ) [26], and the Intuitive Eating Scale-2 [27]. Participants were introduced to hunger training and taught to measure their blood glucose. After the first day of hunger training, the researcher telephoned the participant to ensure that the instructions were understood and able to be followed, and to answer any questions. At the second visit participants had the opportunity to ask questions and talk about any challenges or successes. If participants had difficulty only eating when their blood glucose was 4.7 mmol/L or less in the first week, they wore a continuous glucose monitoring system (CGMS) (iPro2 Continuous Glucose Monitoring System, Medtronic, California, USA) for the second week of the study. The CGMS allowed us to observe blood glucose variation in greater detail than the intermittent finger prick glucose tests. The sensor was inserted subcutaneously into the abdominal area and monitored glucose in the interstitial fluid every five minutes for seven days. On the last visit, all participants had their weight measured again, and were asked about their experience of the study during a semi-structured interview, which was recorded (Philips Voice Tracer digital recorder LFH0622) and transcribed. The exit interviews were examined with a data-driven thematic analysis.

### Feasibility criteria

*A priori* we specified that hunger training would be considered feasible if all of the following criteria were met:

1. 85 % or more of participants completed the study.
2. The average within-person proportion of eating occasions where blood glucose was measured was more than 80 %.
3. The average within-person proportion of eating occasions where blood glucose was below the cut-off was more than 75 %.

We were also interested in examining the within-person correlation between hunger and blood glucose, and body weight change (if any) in overweight participants over the two weeks.

### Analyses

Descriptive statistics were used to answer the feasibility criteria, with adherence measures and the correlation between hunger and blood glucose calculated for each participant before generating within-person point and interval estimates. Study completion was calculated as the number of participants who attended the final visit at day 14 divided by the number of participants who attended the first visit. Adherence to measuring blood glucose was calculated by dividing the number of reported eating occasions where blood glucose was noted by the total number of eating occasions for each participant. Adherence to eating when below the specified blood glucose cut-off was calculated by dividing the number of reported eating reported eating occasions where blood glucose was below the assigned cut-off by the total number of eating occasions with a noted blood glucose value. Differences between groups were analysed using t-tests, with unequal variances where indicated. Paired t-tests were used to compare the average of the first two days of fasting glucose with fasting glucose for the 14-day period.

As no formal power calculations are undertaken in feasibility studies, sample sizes should be based on estimating feasibility outcomes (Arain, Campbell, Cooper, & Lancaster, 2010). We estimated that a minimum of 25 participants was appropriate to estimate the retention rate and adherence to the hunger training intervention. Statistical analysis was performed using Stata 12 (StataCorp, College Station, TX).

## Results

Recruitment took place on July 2, 2014 by sending an invitation email to staff and students at the University of Otago in Dunedin, New Zealand. All participants were recruited in a single day. The pilot study took place from July 8 to Aug 6, 2014 and 30 participants were recruited. One participant discontinued hunger training due to fasting glucose levels being above the cut-off for possible diabetes diagnosis (>7 mmol/L), resulting in 29 participants being included in the final analysis.

The participants were predominantly well-educated, white women, with an average BMI of 31 kg/m^2^ (Table 1). As there were no significant differences in demographic characteristics at baseline between cohorts A and B (data not shown), only the combined results are presented.

**Table 1:** Characteristics of participants

Variable	All (*N* = 29)
Age (years)	43.3 ± 12.5
Height (m)	1.7 ± 0.1
Weight (kg)	89.8 ± 25.2
Body mass index (kg/m^2^)	31.2 ± 9.0
Women, n (%)	22 (76 %)
White ethnicity, n (%)	27 (93 %)
Partnership status, n (%)	
Partnered	16 (55 %)
Non-partnered	13 (45 %)
University degree, n (%)	19 (66 %)
Intuitive eating questionnaire	
Overall score^a^	3.1 ± 0.5
Unconditional permission to eat	3.3 ± 0.6
Eating for physical rather than emotional reasons	2.9 ± 0.7
Reliance on hunger	2.9 ± 0.7
Body-food choice congruence	3.4 ± 0.9
Physical activity (MET-minutes/week)^b^	2019 ± 2955
Hours of sitting per day^b^	7.1 ± 2.6

Note: Unless indicated, values are mean ± SD

^a^The intuitive eating score ranges from one to five, with higher scores indicating greater levels of intuitive eating

^b^median ± IQR

### Adherence

Adherence was measured in terms of overall study retention, glucose measurement prior to eating, and compliance to the blood glucose cut-off. There was no difference in any adherence measures between lean and overweight participants (data not shown). Retention was high, with 28 out of 29 participants (97 %) completing the pilot study, well above our predetermined criterion for determining success of 85 %.

In terms of adherence to the blood testing protocol, the within-person proportion for measuring blood glucose before eating was 94 % (95 % CI 91, 98) of eating occasions, with no significant difference between cohorts A and B. This level of adherence considerably exceeded our *a priori* requirement of measuring before 80 % of eating occasions.

#### Adherence to eating with protocol A

**Fig. 3:**
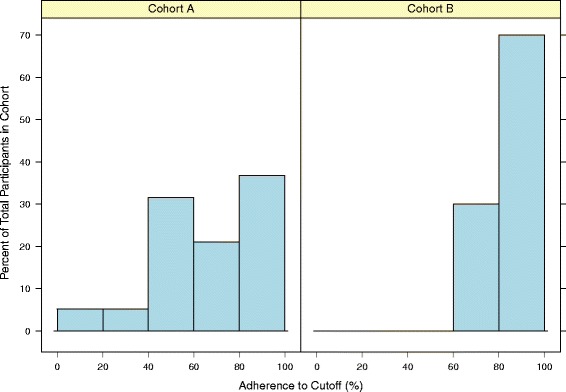
Histogram of adherence to eating below the blood glucose cut-off in cohort A and cohort B

Participants following protocol A, which used a universal blood glucose cut-off of 4.7 mmol/L, adhered to the goal of eating only when blood glucose was below this level 66 % of the time, which was below our within-person adherence requirement of 75 % (Table 2). Furthermore, four out of the nineteen (21 %) participants in this cohort adhered to protocol A on less than half of their eating occasions (Fig. 3), with one participant only adhering on two eating occasions over the two week period (5 % of all eating occasions).

**Table 2:** Adherence to hunger training

Adherence	Cohort A (*N* = 19)	Cohort B (*N* = 10)	*p-value*
Measuring glucose	92.3 % (88.6, 96.0)	96.1 % (93.9, 98.2)	0.149
Eating below glucose cut-off	66.4 % (54.2, 78.6)	84.4 % (78.0, 90.8)	0.010

Data presented as within-person mean (95 % CI)

**Fig. 4:**
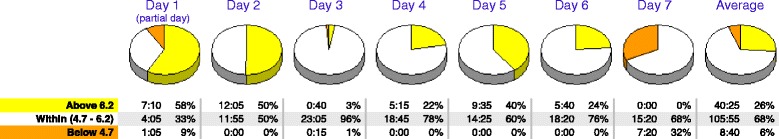
Results from the continuous glucose monitoring sensor from a participant over 7 days, comparing the amount and percentage of time the participant was below the protocol A cut-off of 4.7 mmol/L and protocol B individualised cut-off of 6.2 mmol/L

Of these 19 participants, six who appeared to be struggling with only eating when their blood glucose was 4.7 mmol/L or less during the first week of training wore a continuous glucose measurement sensor during week two. On average, their blood glucose was above the protocol A cut-off of 4.7 mmol/L 85 % of the time over the seven days, or 143 hours of the total 168 hours captured. Additionally, five of these six participants had at least one day where their blood glucose never dropped below 4.7 mmol/L. Fig. 4 illustrates the results from one of these participants, showing their blood glucose over 7 days and the amount of time it was under both protocol A (universal 4.7 mmol/L cutoff) and protocol B (individualised cut-off of 6.2 mmol/L for this participant).

### Adherence to eating with protocol B

*A priori* we decided that the blood glucose cut-off would be deemed feasible if the within-person proportion of measured blood glucose was below the cut-off on more than 75 % of eating occasions. As protocol A was below this requirement, we switched to protocol B for the remaining ten participants. For protocol B participants, their individualised blood glucose cut-off was calculated from the average of fasting glucose over the first two days of hunger training, which resulted in an average cut-off of 5.5 mmol/L (SD 3.0). The use of this individualised protocol increased adherence to the eating protocol to a within-person proportion of 84 % of the time, which was a significant increase over protocol A (*p* = 0.010) and met our benchmark for feasibility.

We examined whether the average of the first two days of fasting glucose was a suitable estimate of the average fasting glucose throughout the two-week period, and therefore was suitable to determine the individualised cut-off. Fasting glucose means were comparable (difference 0.07 mmol/L), with no significant difference between these time periods (*p* = 0.415).

### Correlation between hunger and blood glucose

A significant, albeit modest, inverse within-person correlation was observed between perceived hunger and blood glucose concentrations of r = −0.23 (95 % CI −0.15, −0.31), *p* < 0.001. Cohort B tended to have a stronger correlation between hunger and glucose (r = −0.33 for cohort B vs r = −0.18 for cohort A) although our small numbers probably preclude statistical significance (*p* = 0.082).

### Weight loss

Overweight participants following both protocols achieved significant weight loss over the two-week period, with an average loss of 1.5 kg (95 % CI 2.2, 0.9) and a corresponding reduction in BMI of 0.6 kg/m^2^ (95 % CI 0.3, 0.8), *p* < 0.001 (Table 3). By contrast, lean participants maintained their weight (*p* = 0.337, data not shown). Although participants following protocol B appeared to lose more weight than those in protocol A, differences were not significant (Table 3).

**Table 3:** Change in weight and BMI over 2 weeks in overweight participants

Variable	Cohort A (*N* = 13)	Cohort B (*N* = 9)	*p*-value
Weight change (kg)	−1.1 (−0.5, −1.8)	−2.1 (−0.6, −3.6)	0.134
BMI change (kg/m^2^)	−0.4 (−0.2, −0.6)	−0.8 (−0.2, −1.4)	0.137

Data presented as mean (95 % CI)

### Exit interviews

The major themes that emerged from the exit interviews were: awareness of non-hungry eating, change of dietary intake, and the use of blood glucose monitoring.

#### Awareness of non-hungry eating

Participants reflected that hunger training made them realise that they were used to eating for reasons other than hunger, including as a way to stave off boredom, to cope with emotions, and because of habit:

- It certainly does make you more aware–it was a good thing to do. It cut down a lot of my night-time snacking, just cruising past and something goes in my mouth without thinking. That’s my major problem, this night-time grazing.
- It’s well up there because it’s something that I don’t really pay attention to. I’m just an automatic feeder. Needing to stop and think before I put something in my mouth was really good. And I very seldom eat when I’m hungry. I’m an emotional eater: bored, tired, etc.…I really want to stop doing that.

#### Change of Dietary Intake

Most participants reduced or eliminated snacks:

- I stopped eating morning tea–just a habit because everyone else has something to eat. But I mean, I have an office job–I’m just sitting on my bottom not burning up much energy, so now I just have coffee instead. By lunchtime now, I’m feeling hungry.
- The thing I cut out was the snacking–that was the main impact. For instance I used to eat bag of chips for no reason, just because it seemed like a good idea.

Many participants chose better food, because they were more conscious of their food intake or because they noticed how their blood glucose reacted afterwards:

- My lunches I’ve changed totally. It’s now three mandarins, a pottle of yoghurt, and a banana. And before? It was anything that I wanted.
- I’m not eating things like chips and crackers. Now it’s a treat. It’s really changed how much processed food and I’m definitely eating much more vegetables, and enjoying them. It just seems like a waste now to eat a bag of chips, since then I can’t have dinner.

Many participants reduced their portion sizes at meals, and noted that they were surprised that they didn’t need as much food as they had previously assumed, and could no longer imagine eating as much as before the pilot:

- I still served myself the same portions but I couldn’t eat it all. Which is kind of weird for me. I stopped when I had enough. I don’t know if I’m just getting used to eating a bit less or I realise that I didn’t NEED to eat it all. Whereas before I would finish it because it was wasteful. But now I think, “you don’t have to force yourself to eat stuff that you don’t need to.” And that rating of fullness at the end was quite good, because before I would have that too full, gross, feeling. But now thinking about what full feels like and overfull feels like and what not quite full feels like.
- Cutting down on what I was having for breakfast did make a difference to my blood sugar before lunchtime. So just having porridge OR toast rather than porridge AND toast meant that I could have lunch at lunchtime.

Conversely, a few participants reported that their portion sizes increased, since they were hungrier than usual because they were eating less frequently:

- But when I was hungry, I ate MORE. Because I was having to wait until lunchtimes until I could eat, so when I got lunch, I ate more than I normally would have. If I hadn’t eaten until 1 pm, anything that wasn’t tied down wasn’t safe! Once I pricked my finger, I would have my meal and then the chocolate, because I better have the chocolate now else I’ll have to stab my finger again. It eliminates the grazing but increased my portion sizes.

#### The use of blood glucose monitoring

Many participants commented that their blood glucose was an unpredictable measure of their hunger:

- I was having trouble with the [glucose] readings and matching it up with what I was doing, but it certainly made me think about registering whether I was hungry rather than just eating because of routine.
- Super hungry didn’t seem to corresponding to particularly low glucose. I did find it frustratingly inaccurate in terms of measuring my hunger, even though I was much more in touch with my hunger.

However, most participants viewed measuring their blood glucose as a useful behaviour for gaining awareness of their eating habits:

- It’s not just the fact that you inflict pain on yourself – it’s the fact that you inflict pain and it might say “no” anyway. Really have to think, “look, am I actually feeling hungry enough?”. I think it’s extremely effective since it just makes you more aware. Even if I didn’t hurt, it makes you aware of those wee niggle, but “I’ve only eating a little awhile ago”. The pain element is useful and the fear of the rejection after the pain.
- The psychological thing of having to prick your finger every time you want to eat is a bit of a red herring but it’s quite a relevant thing. I think “hmm, I would like some afternoon tea but my fingers are a bit sore today…maybe I don’t need it”

Many participants commented that the pain with finger pricking reduced after the first week:

- I have the feeling that stabbing gets better. At the beginning it hurt more, but I really can’t feel it anymore.
- I don’t find it particularly onerous. It’s easy enough to fit and only a minor irritation, to prick your finger.

## Discussion

The idea that using finger prick blood glucose monitoring to train individuals to eat only when hungry appeared to be a promising method for weight management, based on the results from a group of researchers [12]. However, neither this original study, nor an offshoot from these authors [28] reported on adherence, a critical consideration for determining success. Thus we repeated the Ciampolini protocol (protocol A) using an adaptive design, which allowed us to determine if an alternative blood glucose cut-off might be more suitable. In fact, we found that only our adaptive version of hunger training (protocol B) was feasible, in terms of meeting all three criteria: study retention, adherence to measuring blood glucose, and adherence to eating below the blood glucose cut-off. It is difficult to compare our findings as neither of the previous studies reported on feasibility per se. However, both previous studies reported retention of 80 % of participants [13, 28], which is likely comparable to our retention rates, considering their longer duration.

We anticipated that adherence to measuring blood glucose prior to every eating occasion would be an issue, however this did not seem to be the case. Participants measured their blood glucose before nearly every meal over this two-week trial. Our qualitative data suggests that participants thought that the pain and hassle of pricking their fingertips and measuring their blood glucose was not as bad as they had originally expected. Although our adherence to measuring blood glucose was considerably higher than that reported with diabetics [15], this is likely to be influenced by the short-term nature of our trial.

While both the original (protocol A) and adapted (protocol B) protocols achieved our criteria for success of feasibility in terms of retention and adherence to measuring blood glucose, only the protocol B met the criterion for adherence to eating below the blood glucose cut-off. Participants’ difficulty of adhering to only eating when their blood glucose was under 4.7 mmol/L is consistent with large surveys that show that this concentration is below the fasting glucose level for the majority of non-diabetic adults [16, 17]. Thus adapting our protocol to use an individualised blood glucose cut-off substantially improved adherence to only eating when blood glucose was below the cut-off. Furthermore, this adaptation might improve weight loss success as well, at least over the short-term. Our overweight participants lost 1.5 kg on average over the fortnight, with those following protocol B losing nearly twice as much weight compared with protocol (although this difference did not reach statistical significance). This finding was intriguing given we anticipated that the more lenient cut-off (protocol B) would diminish rather than enhance weight loss. However, both the diary information and the accompanying qualitative interviews showed that protocol B was more successful at encouraging participants to only eat when hungry. One limitation of our data is that although we requested brief dietary information as part of the two-week diary, this was not detailed enough to allow energy intake to be calculated. Thus exactly how this more lenient cut-off changed dietary intake is uncertain. Furthermore our trial only lasted two weeks and longer follow-up is required to determine whethe*r* hunger training offers a viable method for sustained weight loss. As hunger training will be one support strategy in the upcoming SWIFT trial, we will have the opportunity to examine the effectiveness of hunger training on weight loss over two years. However, our findings do support those of Ciampolini et al. who demonstrated an average weight loss of 5.8 kg over 5 months in overweight participants [13].

Our findings of only a weak correlation between hunger and blood glucose are in agreement with the range of results reported in the literature [18, 19]. The weak and inconsistent association between hunger and blood glucose was the most problematic element for participants, as evident in their feedback. However, most participants felt that measuring blood glucose still provided valuable feedback and was crucial for modifying their eating behaviour, irrespective of its limitations. It seems as if the benefits of objective biofeedback provided by measuring their blood glucose levels enhances the benefits of monitoring appetite [29, 30] and dietary intake [31]. Similarly, self-monitoring of blood glucose in diabetics has been shown to improve adherence to nutritional recommendations and decrease body weight [32]. Furthermore, the necessity to measure blood glucose concentrations required participants to record their appetite and blood glucose results at that instant, rather than hours later, which may be beneficial as a shorter time interval between eating and dietary self-monitoring has been shown to be significantly associated with weight loss [33].

The main strength of this feasibility study is our careful measure of adherence, which has not previously been described for this intriguing weight management strategy. Feasibility and pilot studies are an important step before a large trial, and are especially useful when feasibility objectives and success criteria are defined *a priori* [34]. Including an adaptive design approach helped us to efficiently find the most suitable blood glucose cut-off for our participants, which appears to be an improvement over the original method. This study provided us with confidence about the feasibility of the hunger training method in general, and knowledge that an individualised blood glucose cut-off is the most viable approach. Hunger training may provide an effective strategy for weight loss by teaching people to eat according to their physical hunger rather than in response to environmental, social, or emotional cues. Based on our findings, the SWIFT trial will include our adapted version of hunger training as one of four intervention arms [14]. However, our study also has some limitations. As befitting a feasibility study, it was relatively short, but was based on the original protocol that showed the majority of participants were trained after two weeks. While it would have been interesting to use continuous monitoring in all participants, it was cost prohibitive. However, with the advancements in non-invasive glucose monitoring, such as using contact lenses [35] and temporary tattoos [36], continuous (and pain-free) blood glucose monitoring is likely to become more affordable and accessible.

## Conclusions

Our results show that participants are willing to participate in hunger training, measure their glucose before eating, and only eat when their blood glucose is under their individualised cut-off, at least in the short-term. The results of our study suggest that the adapted version of the hunger training protocol is feasible, and viable to use as a support strategy in the SWIFT trial. Further testing of hunger training in the SWIFT trial will allow us to examine the effects of this method over a two-year duration in combination with healthy eating and exercise advice.

## Abbreviations

BMI: Body mass index
CGMS: Continuous glucose monitoring system
CI: Confidence interval
IQR: Interquartile range
MET: Metabolic equivalent of task
SD: Standard deviation
